# Notch Signaling in Central Nervous System: From Cellular Development to Multiple Sclerosis Disease

**DOI:** 10.2174/1570159X22666240731114906

**Published:** 2024-07-31

**Authors:** Hamid Askari, Fatemeh Rabiei, Masoomeh Yahyazadeh, Giuseppe Biagini, Maryam Ghasemi-Kasman

**Affiliations:** 1 Student Research Committee, Babol University of Medical Sciences, Babol, Iran;; 2 Department of Biomedical, Metabolic, and Neural Sciences, University of Modena and Reggio Emilia, Modena, Italy;; 3 Cellular and Molecular Biology Research Center, Health Research Institute, Babol University of Medical Sciences, Babol, Iran;; 4 Department of Physiology, School of Medicine, Babol University of Medical Sciences, Babol, Iran

**Keywords:** Multiple sclerosis, notch signaling, microglia, astrocytes, oligodendrocytes, neurogenesis

## Abstract

**Introduction/Objective:**

Multiple sclerosis (MS), is characterized by autoimmune-driven neuroinflammation, axonal degeneration, and demyelination. This study aimed to explore the therapeutic potential of targeting Notch signaling within the central nervous system (CNS) in the context of MS. Understanding the intricate roles of Notch signaling could pave the way for targeted interventions to mitigate MS progression.

**Methods:**

A comprehensive literature review was conducted using databases such as PubMed, Web of Science, and Scopus. Keywords such as “Notch signaling,” “neuroglial interactions,” and “MS” were used. The selection criteria included relevance to neuroglial interactions, peer-reviewed publications, and studies involving animal models of MS.

**Results:**

This review highlights the diverse functions of Notch signaling in CNS development, including its regulation of neural stem cell differentiation into neurons, astrocytes, and oligodendrocytes. In the context of MS, Notch signaling has emerged as a promising therapeutic target, exhibiting positive impacts on neuroprotection and remyelination. However, its intricate nature within the CNS necessitates precise modulation for therapeutic efficacy.

**Conclusion:**

This study provides a comprehensive overview of the potential therapeutic role of Notch signaling in MS. The findings underscore the significance of Notch modulation for neuroprotection and remyelination, emphasizing the need for precision in therapeutic interventions. Further research is imperative to elucidate the specific underlying mechanisms involved, which will provide a foundation for targeted therapeutic strategies for the management of MS and related neurodegenerative disorders.

## 1 INTRODUCTION

Multiple sclerosis (MS) is a neurodegenerative disease of the central nervous system (CNS) characterized by autoimmune processes. This leads to the gradual development of deficits due to the degeneration of axons in the CNS caused by demyelination [1]. Two potential strategies for preventing axonal loss include mitigating inflammatory attacks in the CNS and repairing myelin. Consequently, therapeutic interventions that specifically address several signaling pathways implicated in neuroprotection and remyelination exhibit promising promise in surmounting the obstacles encountered during the advancement of treatments for MS [2].

The Notch pathway represents one of the targets under consideration. The involvement of Notch signaling in the differentiation and functionality of many cell types that play essential roles in the formation and clinical advancement of MS has been suggested [3]. Many cell types, including neural stem cells (NSCs), neurons, oligodendrocytes (OLs), astrocytes, and microglia, express Notch, demonstrating its special function in the CNS. Significantly, inflammatory/demyelinating lesions observed in MS and corresponding animal models have a substantial presence of Notch receptors, their ligands, and downstream activation targets [4]. Numerous investigations are currently focused on the Notch pathway and its associated variables that interact with or affect this system to identify a viable therapeutic intervention for this particular disease [5].

We provide a comprehensive overview of the intricate interplay between Notch signaling and various glial cell types in the CNS, shedding light on its diverse functions in neuroinflammation, neurodegeneration, and myelination. To achieve this goal, we conducted a thorough search of relevant literature using keywords such as “Notch signaling”, “neuroglial interactions”, “microglia”, “astrocytes”, “oligodendrocytes”, “oligodendrocyte progenitor cells”, and “neurogenesis” in databases including PubMed, Web of Science, and Scopus. The selection criteria for the reviewed articles included relevance to Notch signaling in neuroglial interactions, publication in peer-reviewed journals, and a focus on studies involving animal models of MS and therapeutic approaches in MS.

## 2 NOTCH SIGNALING

In 1914, through the works of John S. Dexter and, subsequently, Thomas H. Morgan, multiple mutant alleles were found for the heritable phenotype of notched wings in the fruit fly *Drosophila melanogaster.* Thus, the name “Notch” for the gene seemed to fit. The Notch signaling pathway has recently become an essential mechanism underlying intercellular communication [4].

The Notch signaling pathway is pivotal for diverse processes, such as cell activation, proliferation, cell fate, differentiation, and apoptosis [6]. In contrast to the classical signaling pathways, which typically involve a series of defined steps, the receptors in the Notch signaling pathway undergo three cleavages and are then translocated into the nucleus without any intervening steps [7]. There are four types of Notch receptors, all of which are heterodimeric, single-span transmembrane glycoproteins [8]. Notch-1, -2, -3, and -4 are encoded by genes on human chromosomes 9, 1, 19, and 6, respectively [9]. Both Notch-1 and Notch-2 are found in a wide variety of tissues. However, Notch-3 is highly expressed in vascular smooth muscle and pericytes, while Notch-4 is mainly localized on the endothelium [10].

Following transcription and translation, Notch precursors are synthesized in the endoplasmic reticulum (ER), where they are glycosylated at the tandem EGF-like repeat domain and subsequently transported to the Golgi [11]. The Golgi apparatus undergoes the first of many proteolytic processes, S1 cleavage, which is carried out by a furin-like protease. This process converts Notch to its mature heterodimer [8]. The Notch receptor comprises an intracellular domain (NICD), an extracellular domain (ECD), and a transmembrane domain. The ECD contains 29 to 36 EGF-like repeats and a negative regulatory region (NRR), containing three cysteine-rich Lin12-Notch repeats (LNRs) and a heterodimerization region [9, 12]. The NRR is essential for preventing improper receptor activation. The activation of Notch occurs through contact between the 11th and 12th EGF repeats of the receptor and the DSL (Delta/Serrate/Lag-2) domain of the ligand located on the nearby cell [13]. There are five recognized Notch ligands in humans, each with distinct roles: delta-like ligand 1 (DLL1), delta-like ligand 3 (DLL3), delta-like ligand 4 (DLL4), Jagged-1 (JAG1), and Jagged-2 (JAG2) [10]. Notch ligands share certain structural similarities with their receptors; transmembrane proteins, which are composed of EGF-like repeats, are regulated by the processes of ubiquitylation and endocytosis [8]. In the inactive state, also known as the “autoinhibited conformation”, the S2 site is shielded by the LNR domain [14]. Upon ligand attachment, the pulling force exerted exposes the cleavage site for the ADAM10 protease to create the Notch extracellular truncation (NEXT) region, which comprises the NICD and the transmembrane domain [15]. The γ-secretase complex catalyzes the further cleavage of NEXT at its S3 site, liberating the NICD, which is transported to the nucleus *via* the endosomal route [16]. S3 cleavage occurs both on the cell membrane and in the endosome after NEXT has been endocytosed. These two possibilities have been referred to as endocytic-activation and endocytosis-independent models [17].

The NICD contains an RBPJ association module (RAM) domain, which interacts with transcription factors and comprises seven ankyrin repeat (ANK) domains. Conserved motifs (PEST domains), located at the very end of the NICD, include degradation signals and are, therefore, essential for protein stability [8]. NICD converts the transcriptional repressor CSL (CBF1, Suppressor of Hairless; Lag1, also known as Retinoic acid-binding protein kinase (RBPJ)) into an activator by attaching to CSL in the nucleus [18]. The association between the NICD and CSL is sustained by the protein Mastermind-like (MAML), and the ternary complex composed of the Notch ICD, MAML, and CSL is responsible for activating the expression of downstream genes [13]. Fig. (**1**) provides an overview of the Notch signaling pathway.

Proteins in the nucleus, cytoplasm, and extracellular space regulate Notch signaling. Notch receptors are structurally and functionally diverse, influencing and being influenced by one another. Hence, multiple cell fates may be determined by distinct combinations of Notch receptors activated by the same ligand or by a single Notch receptor activated by various ligands [19].

## 3 NOTCH SIGNALING IN GLIAL CELLS

### 3.1 Notch Signaling in Microglia

There have been extensive studies on the role of the Notch signaling pathway in the CNS. It is significant among other known brain development regulatory pathways. Considerable evidence suggests the involvement of Notch signaling in the etiology of CNS pathologies, which also affects postnatal development [20]. The special function of Notch in the CNS involves its widespread expression in cell types ranging from NSCs to neurons, OLs, astrocytes, and microglia. Although much is known about the function of Notch in the aforementioned CNS cells, such as its crucial roles in neuronal development, synaptic plasticity, and gliogenesis, knowledge of its expression and functions in microglia, the resident immune cells of the central nervous system, is still lacking.

Microglia are resident immune cells that serve as antigen-presenting cells in the CNS [21] and are known as dynamic macrophages that play a functional role in clearing dead cells during both normal and pathological cellular development [22]. Many studies have implicated microglia in the pathogenesis of CNS and neuroinflammatory diseases [23].

#### 3.1.1 Microglial Development and the Notch Signaling Pathway

In the brains of neonatal rats at three postnatal days, OX-42 or lectin labeling revealed the presence of the Notch-1 receptor on microglia. Intriguingly, colocalization of JAG1 and DLL1 has been observed in amoeboid microglia (AMCs) located within the subventricular and corpus callosum areas. Considering the compact arrangement of microglia within cell clusters, it is plausible that Notch ligands function in a paracrine and autocrine manner, influencing their surrounding microenvironment. Surprisingly, the functional expression of Notch signaling in AMCs is observed in postnatal rats at an early stage [24].

A significant decrease in Notch-1 receptor expression was noted in rats from postnatal day 1 to postnatal day 14, adding an intriguing aspect to our findings. Furthermore, western blot analysis of the corpus callosum revealed a concurrent decrease in the level of the Notch-1 protein. This decrease is in comparison to that in the early postnatal stage, and it correlates with the downregulation of Notch-1 expression in microglia. These compelling data support the idea that AMCs expressing the Notch-1 receptor play a crucial role in maintaining a population of embryonic or immature microglia during early brain development. Moreover, the consistent production of Notch-1 and its ligands by early postnatal AMCs suggests a potential link between Notch control and AMC function in CNS development [24].

#### 3.1.2 Microglial Activation and the Notch Signaling Pathway

Recent research has suggested that the Notch pathway plays a significant role in neuroinflammation disorders by regulating microglial function [25]. For instance, the Notch pathway has been implicated in MS, where inhibiting the pathway using γ secretase inhibitors (GSIs) reduced demyelination and paralysis in MS animal models [26]. This highlights the crucial involvement of microglia, as Notch pathway modulation influences the pathological processes associated with MS.

Further investigations have focused on exploring the molecular and cellular changes in neuroinflammatory diseases dependent on the Notch signaling pathway. Studies have indicated that OX-42-positive small cells, likely microglia, play a role in regulating Notch-1 expression in demyelinated lesions exposed to chemical induction [27]. The researchers used cultured microglia derived from rats and murine BV-2 cells. These cells were subjected to lipopolysaccharide (LPS) treatment. The results of the trials revealed a concurrent increase in the expression of Notch-1 and JAG1 mRNA, whereas the expression of DLL1 decreased.

Furthermore, researchers have revealed the synchronized control of Notch-1 signaling and inflammation in microglia treated with LPS. This control mechanism is facilitated by the RAS pathway in combination with gastrodin [28]. Notably, gastrodin efficiently suppresses the expression of crucial constituents of the Notch signaling pathway implicated in the activation of microglia, such as Notch-1, NICD, RBPJ, and hairy and enhancer of split-1 (Hes-1) [25].

Moreover, in postnatal rats, LPS injection increased Notch-1, TNF-α, and IL-1β immunofluorescence in AMCs. In addition, treatment with DAPT, an inhibitor of Notch cleavage, reduced the levels of IL-1β and TNF-α but surprisingly had the opposite effect on Notch-1 labeling. Notably, DAPT was found to potentially suppress microglial activation and the production of proinflammatory cytokines in the spinal cord, in addition to the overexpression of the Notch pathway [29]. These collective insights provide a comprehensive understanding of the intricate interplay between Notch signaling and neuroinflammation, offering potential avenues for future therapeutic interventions in the context of microglia-related diseases.

A study reported the expression of Notch receptors in microglia and confirmed the functional activity of Notch signaling during inflammatory activation. However, in the context of inflammatory activation, the transcription of Hes-1, a direct target gene of Notch transcriptional repressors, is downregulated. Surprisingly, when the transcription of Notch-1 is inhibited in microglia, it leads to elevated levels of proinflammatory cytokines. Conversely, stimulation of Notch signaling through the ligand JAG1 decreases proinflammatory cytokine and nitric oxide (NO) production while enhancing activated microglial phagocytic activity [30]. Such inconsistent findings may arise from potential interactions between Notch signaling and other intracellular pathways, suggesting that complex interactions within the cellular signaling network could contribute to the observed discrepancies. Our analysis of these findings suggested that the role of Notch signaling in microglia is context-dependent and multifaceted. While Notch signaling may exacerbate neuroinflammation under certain conditions, such as when Hes-1 transcription is inhibited, it may also have anti-inflammatory effects, as observed with JAG1 stimulation. These contrasting observations underscore the complexity of Notch signaling in microglia and highlight the need for further in-depth investigations to unravel the underlying mechanisms governing these diverse cellular responses in neuroinflammation. Fig. (**2**) shows a summary of the role of microglial activation in demyelination.

### 3.2 The Notch Signaling Pathway in Astrocytes

Neurons are the first cells created from neural precursor cells throughout mammalian nervous system development. Committed neuronal precursors and immature neurons activate Notch signaling, which in turn causes astrocyte-specific genes to be demethylated and activated through the JAK-STAT pathway as regulated by cytokines, helping the remaining neural progenitors develop into astrocytes [31]. Astrocytes have a high level of Notch signaling activity, and their inhibition is sufficient to initiate a neurogenic program [32]. Additionally, emerging evidence suggests a role for Notch signaling in regulating inflammatory activities in astrocytes. Although the exact underlying mechanisms remain under investigation, studies have indicated that Notch signaling may modulate the expression of inflammatory mediators and cytokines in astrocytes, thereby influencing neuroinflammatory responses within the CNS [33]. Further research is warranted to elucidate the precise role of Notch signaling in regulating inflammatory activities in astrocytes and its implications for neurodegenerative diseases such as multiple sclerosis.

Modifications in the proportion of neurons to glial cells generated throughout development can substantially influence neural activity. Studies have shown that neurons are typically generated before glial cells, suggesting that the emergence of fledgling neurons has the potential to affect neural progenitor cell (NPC) differentiation and proliferation by participating in regulatory mechanisms [34].

Throughout development, neural differentiation into glial cells is governed by a complex interplay of internal mechanisms and extracellular signals [35]. The circumferential environment plays a crucial role in regulating this transition, as evidenced by studies showing that NPCs cultivated on embryonic cortex slices generate neurons. In contrast, on postnatal slices, they generate astrocytes [36]. Notably, astrocytic differentiation is stimulated by IL-6 family cytokines, such as ciliary neurotrophic factor (CNTF), leukemia inhibitory factor (LIF), and cardiotrophin-1 (CT-1), which activate the JAK-STAT signaling pathway. CT-1, released from embryonic neurons, was identified as a critical component promoting the NPC shift from neurogenesis to astrocytogenesis *in vivo*, while other IL-6 family cytokines were suggested to promote glial cell formation [37]. Interestingly, the receptivity of NPCs to external cues is influenced by intracellular processes, such as CpG promoter methylation, which reduces STAT binding and early gliogenesis in response to gliogenic cytokines such as CT-1 in glial-specific progenitors [38]. Although several pathways have been shown to contribute to glial growth, the precise interplay and coordination between these routes remain a topic of ongoing investigation.

A previous study revealed a link between Notch signaling, cytokine signaling, and the demethylation of glial-specific promoters [34]. One study revealed that during midgestation, JAK-STAT signaling activators can trigger NPC differentiation into astrocytes. However, in NPCs at earlier developmental stages, Notch signaling is required prior to their differentiation into astrocytes. By coculturing E11.5 NPCs with embryonic cortical neurons, researchers successfully triggered astrocytic differentiation by activating Notch signaling. Notch signaling has been shown to activate STAT3 binding site demethylation in the glial fibrillary acidic protein (GFAP) and S100β promoters. This observation shows that Notch signaling facilitates the demethylation of gene promoters involved in astrocytic development. The use of LY411575 to suppress Notch signaling *in vivo* led to a reduction in astrocyte differentiation and an increase in the methylation of the GFAP promoter.

An examination of the ventricular zone (VZ) of the cortex at E11.5 revealed that cells close to neurogenin-1 (NGN-1)-expressing cells exhibited robust activation of Notch signaling. A mutually exclusive relationship was observed between NGN-1 expression and the induction of Notch signaling. In addition, NGN-1-expressing cells also exhibited concurrent expression of the Notch ligands JAG1 and DLL1. These findings strongly indicate that early postmitotic neurons and neuronal committed precursors significantly stimulate Notch signaling in nearby precursor cells [32].

In the absence of Notch signaling, methylated promoters prevent cytokine-activated STATs from binding, guaranteeing that early-stage glial differentiation is avoided despite the presence of gliogenic cytokines [37]. Premature astrocytic maturation in E11.5 precursors does not seem to be driven by active Notch signaling alone *in vivo* but rather by a combination of factors, including NFIA and cytokine overexpression [39]. Further research involving genetic techniques, such as the deletion of Notch ligands from cells expressing NGN1, could identify suitable alternatives. However, *in vivo* investigations have not yet explored the primary source of Notch-activating signals.

### 3.3 The Notch Signaling Pathway in Oligodendrocytes

OLs predominantly constitute myelin in the CNS. O-2A progenitors (from which OLs originate) are migratory and proliferative OLPs. Current research indicates that OLPs are formed in very confined areas of the CNS and then travel broadly across the white matter of the CNS. In this section, we propose that the development of region-specific OLs is regulated by a delicate balance between differentiation-promoting signals originating from the ventral neural tube and inhibitory signals originating from the dorsal neural tube [40]. In addition, we examined new studies that provided more information on the intracellular Notch pathways that may govern OL differentiation.

#### 3.3.1 Timing Controls the Differentiation and Myelination of OLs

Signals that interact with receptor molecules on the surface of OL cells significantly affect OL differentiation and maturation timing, with the Notch pathway being a key determinant in this process [41]. During the development of the rat optic nerve, both OLs and OLPs express Notch-1 receptors. Concurrently, JAG1 is expressed in the axons of retinal ganglion cells, but its expression decreases as myelination progresses. Laboratory experiments have shown that the differentiation of OLs can be hindered by the use of JAG1 or a soluble form of recombinant DLL1. Therefore, the reduction in JAG1 expression in the optic nerve might stimulate the differentiation of OLs and/or myelination of axons. This decrease in JAG1 expression could be associated with the neural activity of retinal ganglion cells as the eyes open.

Additionally, OPC transplantation has shown promising results in ameliorating motor and sensory impairment in rats with spinal cord contusion (SCC) by increasing myelin synthesis, which may be related to shifts in the production of Notch signaling-related proteins, such as Notch receptors and ligands, within the microenvironment of the injured spinal cord. Specifically, it is hypothesized that transplanted OPCs influence the local microenvironment, leading to alterations in Notch signaling. This can result in a shift toward promoting OPC differentiation and remyelination, ultimately contributing to functional recovery. The observed improvements in motor and sensory function following OPC transplantation may be attributed, at least in part, to changes in Notch signaling-related proteins, which promote remyelination and axonal integrity [42]. These findings underscore the complex and dynamic role of Notch signaling in OL development and myelination, providing valuable insights for potential therapeutic interventions to promote myelin repair in neurodegenerative disorders.

#### 3.3.2 Notch Signaling in Oligodendrocyte Growth and Transcriptional Regulation

The understanding of the intracellular control of OL growth and the regulation of transcriptional processes remains limited despite the identification of intercellular signals responsible for early OL maturation. Notably, impeding OL development by activating the Notch receptor on OPCs or immature OLs has been demonstrated. Upon Notch receptor activation, the NICD of Notch undergoes cleavage, moves to the nucleus, and triggers the Hes family of basic helix-loop-helix (bHLH) transcription factors with the assistance of the Su(H)/RBPJ protein. Consequently, the activation of Notch receptors in OL lineage cells leads to the upregulation of Hes5 [43]. To counteract the effects of proneural bHLH proteins, Hes proteins can form heterodimers with other proteins [44]. Remarkably, certain bHLH genes, such as the mash1 gene, contain an N-box sequence in their promoter regions, potentially serving as a binding site for Hes family proteins [45]. Consequently, during neurogenesis, bHLH proteins that induce differentiation are suppressed by Hes family proteins. These results contribute to the complex understanding of Notch signaling in OL development and have implications for potential therapeutic interventions targeting this pathway.

#### 3.3.3 The Initiation of OLP Specification Relies on bHLH

As previously stated, OL formation is controlled by ventral and dorsal region signals, similar to how neuronal cells are regulated [46]. Notch and its ligands regulate OL lineage cell development. The expression of DLL1 and JAG1 in the ventricles of the CNS suggested that these molecules have complementary domains with adjacent and nonoverlapping boundaries. This suggests that specific combinations of Notch and its ligands may play a role in controlling the formation of different types of neural cells [47]. In the spinal cord sections of the mice, the ventral VZ contained three distinct strips of areas expressing JAG1. One of these areas overlapped with the foci of OLPs both in terms of location and timing [48].

As Hes family genes reside downstream in the Notch signaling pathway, bHLH transcription factors are closely linked to the production of Notch, DLL, and JAG. The correlations observed between NGN-1 and Mash1 expression and between DLL1 and JAG in the mouse CNS raise the possibility of a regulatory connection between bHLH factors and Notch signaling [47]. The combined influence of Notch signaling and the bHLH family is indispensable for the initial stages of OL development. Precise arrangements of these patterning molecules can activate the formation of OLPs in specific regions of the VZ.

### 3.4 The Notch Signaling Pathway in NG2 Cells

Nerve/glial-antigen 2 (NG2) cells are oligodendrocyte progenitors. NG2 cells constitute approximately 4-8% of adult brain cells throughout the gray and white matter [49]. Growth factors can induce NG2 cells to differentiate into astrocytes and neurons [50]. In different CNS regions, OLs continually divide, multiply, and differentiate into adult OLs [51].

NG2 cells may produce OLs, astrocytes, and neurons under pathological conditions [52]. After CNS injury, the Notch signaling cascade in the microenvironment increases the number of astrocytes in NG2 cells [53], and in glial scars, astrocytes dominate [54]. Suppression of the Notch signaling system decreases inflammation-related secondary damage and limits the proliferation of reactive astrocytes [55]. There is hope for neurofunctional recovery after brain damage since NG2 cells may generate new neurons [56]. Notch signaling inhibits neuronal growth. Blocking Notch signaling promotes neuronal development, maturation, and function [57].

#### 3.4.1 Notch Signaling and NG2 Cell Differentiation

CNS cell fate is controlled by the Notch signaling pathway. It also participates in various essential events during the development of glial cells, such as maintaining NG2 cells in an unvaried state.

Notch ligands reduce NG2 cell differentiation, suggesting that the Notch pathway controls this differentiation [41]. *In vivo*, experiments using a mouse model in which Notch-1 was selectively inactivated across the OL lineage showed that Notch-1 signaling enhanced NG2 cell growth but impeded differentiation and myelin production [58]. In the context of older animals, the expression of Notch-1 and JAG1 did not have any significant impact on remyelination. Specifically, the remyelination characteristics of knockout and control Plp-CreER Notch-1(lox/lox) transgenic mice treated with cuprizone were unchanged when Notch-1 was ablated in NG2 cells [59].

The recruitment of Deltex1 to F3/contactin, a Notch ligand, leads to the translocation of the NICD to the nucleus through γ secretase-dependent mechanisms. Inside the nucleus, the NICD/RBPJ/Deltex1 complex induces the expression of myelin-associated glycoproteins, facilitating the differentiation of NG2 cells into mature OLs. Interestingly, this mechanism can be disrupted by certain dominant-negative forms of Notch-1, Notch-2, and Deltex1 mutants lacking the RING-H2 finger pattern. At the same time, the process remains unaffected by dominant-negative RBPJ or Hes1 antisense oligonucleotides [60].

## 4 THE NOTCH SIGNALING PATHWAY AND NEUROGENESIS

The development of the CNS is a multifaceted process that is strictly regulated at both times and places. A significant proportion of neural cells in the brain are generated during the embryonic stage of development. However, it is worth noting that the adult brain possesses limited stem cells. Throughout an individual's lifespan, adult NSCs have the capacity to produce newly formed neurons. The stem cells that are responsible for brain development are known as radial glia, and they are located in the VZ. NSCs are the origin of the neurogenic lineage, and this process is now widely acknowledged [61].

They propagate rapidly, leading to the emergence of intermediate progenitors that subsequently increase the pools of progenitors and neural progeny [62]. According to their birth order, neuroblasts (NBs) travel radially across the cortex, producing layers from the inside out. While projection NBs travel along radial glial fibers to the superficial layers of the pallium, interneuron progenitors in the ganglionic eminence of the subpallium engage in cortex-bound tangential migration across a considerable distance [63].

NSCs are required to undergo division not only to generate specialized progeny but also to maintain the reservoir of stem cells throughout the neurogenic phase of embryonic brain development. It is imperative for at least one of the resulting daughter cells to retain its role as a functional NSC [64, 65].

The subventricular zone (SVZ) lateral wall (LW) and the DG subgranular zone (SGZ) are two prevalent sites within the adult brain where NSCs are commonly found. Neurons are produced by adult NSCs *via* intermediate progenitors/transient amplifying progenitors, which undergo fast cell division to give rise to NBs and neurons in quick succession.

Quiescent NSCs are the most common type; however, active NSCs are sometimes present. NSCs within the mature brain predominantly remain quiescent and exhibit infrequent division. Nonetheless, quiescent NSCs likely transition from active NSCs to quiescent NSCs [64]. In the process of becoming neurons, astrocytes, and OLs, activated NSCs undergo several cell division cycles and produce asymmetrically dividing offspring progenitors [65]. The process of symmetrically dividing active NSCs to yield two NSCs commonly occurs during the initial phases of brain development. However, its occurrence in the adult brain is still a subject of ongoing debate [66]. The role of Notch signaling in modulating NSC equilibrium and differentiation within the context of adult neurogenesis will be the primary topic of discussion in this section.

Both JAG1 and DLL1 are expressed in the adult brain. JAG1 is the primary ligand for Notch signaling in the SVZ [67]. Depletion of JAG1 within the postnatal brain impedes NSC self-renewal, exhibiting similarities to the phenotypic outcomes associated with functional deficiency of the Notch pathway [68]. The DLL1 ligand is present in the brain, albeit at a diminished expression level [67]. Administering DLL4 through infusion into the SVZ resulted in increased NSC proliferation and improved the survival of offspring. Conversely, the targeted inactivation of the DLL1 gene conditionally led to a depletion of quiescent NSCs [69].

On the other hand, contrary to popular belief, DLL3 does not contribute to the preservation of NSCs but rather guides neurons through the differentiation pathway [70]. Notably, some Notch ligand genes, such as DLL1, are repressed at the transcriptional level due to canonical Notch signaling [71].

In addition to the abovementioned receptors and cascades, emerging evidence suggests potential correlations between Notch signaling pathways and nerve growth factor (NGF)/brain-derived neurotrophic factor (BDNF) in the context of neurodegenerative diseases. While our review primarily focuses on the interactions between Notch signaling and glial cell types in the CNS, the relationship between Notch pathways and neurotrophic factors such as NGF and BDNF is an area of increasing interest and relevance. Previous studies have indicated that Notch signaling may influence the expression and activity of NGF and BDNF receptors, thereby modulating neurotrophic signaling cascades involved in neuronal survival, synaptic plasticity, and neurogenesis [72].

Moreover, recent investigations have highlighted potential crosstalk between Notch pathways and NGF/BDNF signaling in regulating glial cell function and neuroinflammation. For instance, Notch signaling has been shown to modulate the expression of NGF/BDNF receptors in microglia and astrocytes, thereby influencing their responses to neuroinflammatory stimuli [73]. Conversely, NGF and BDNF have been reported to regulate Notch signaling activity in glial cells, suggesting bidirectional interactions between these signaling pathways [74, 75].

Furthermore, preclinical studies investigating the therapeutic potential of NGF/BDNF in neurodegenerative diseases have demonstrated that NGF/BDNF cross-regulates Notch signaling pathways, indicating that complex interplay between these signaling networks is involved in disease pathogenesis and potential treatment outcomes [76]. However, further research is warranted to elucidate the precise mechanisms underlying the correlations between Notch pathways and NGF/BDNF signaling and their implications for neurodegenerative disorders such as MS. As a result, Notch signaling is self-regulatory in the niche, with activated cells repressing ligand expression. Neuronal differentiation, survival, and plasticity, which are all linked to the Notch phenotype, are mediated by Notch target genes [77].

The removal of Notch-1 from identical NSC populations had a distinct effect, affecting solely active NSCs while leaving quiescent NSCs unaffected [78], indicating the potential involvement of alternative Notch receptors in maintaining the pool of quiescent NSCs. During development, Achaete-scute homolog 1 (ASCL1) expression is controlled by the Notch-induced transcription factors HES1 and HES5 [79]. Neuronal fate commitment, neural differentiation, and NSC proliferation are influenced by the proneural factor ASCL1. Mediated by ASCL1, dormant NSCs can wake up and start doing their thing [80]. To maintain their quiescent state, NSCs exert precise control over ASCL1 levels by facilitating ASCL1 degradation through the E3 ubiquitin-protein ligase HUWE1 [64].

Unlike immature neurons (Type-3 cells) and induced pluripotent cells (iPSs) (Type-2 cells), radial and horizontal NSCs (Type-1 cells) exhibit notably heightened Notch signaling activity. A significant marker for distinguishing NSCs from other cell types, including proliferative committed progenitors in the DG, is the transcription of the Notch target Hes5 [81].

Hes5, a highly effective indicator of Notch pathway activation, is a direct target of Notch signaling within the CNS [82]. Genetic studies involving the deletion of specific genes have provided substantial evidence for the involvement of Notch signaling components in regulating adult dentate gyrus (DG) neurogenesis. The process of neurogenesis in the adult DG is notably impacted through the targeted removal of Notch-1 *via* conditional deletion, as well as by the heightened expression of activated Notch-1 [83]. Reduced mitotic progenitors and a loss of type 1 cells occur without Notch-1 [84]. Conversely, the expression of NICD, which results in the activation of Notch-1 signaling, induces the expansion of radial NSCs while simultaneously shifting the balance from neuronal generation to the production of glial cells [83]. The genetic removal of Notch-1 resulted in a reduction in the number of active NSCs, yet intriguingly, it did not influence the quantity of quiescent radial cells. This observation is noteworthy, particularly considering the established function of Notch-1 in the SVZ [84].

A decrease in Rbpj gene expression in NSCs results in a temporary increase in the number of iPSCs and NBs. Disrupted adult DG neurogenesis is associated with the initiation of NSC activation due to Rbpj loss, consequently accelerating the decline in the NSC reservoir [85]. Similar to the characteristics observed in the Rbpj conditional knockout phenotype, the postnatal elimination of JAG1 results in a diminished dentate gyrus size, accompanied by a temporary increase in SGZ neurogenesis, followed by the eventual exhaustion of NSCs [86]. Given the shared neurogenic niche among various stem cell populations, Notch signaling serves as a pivotal mechanism not only for governing the role of NSCs within the DG but also as a ubiquitous signaling pathway across diverse cell populations.

**Fig. (3):**
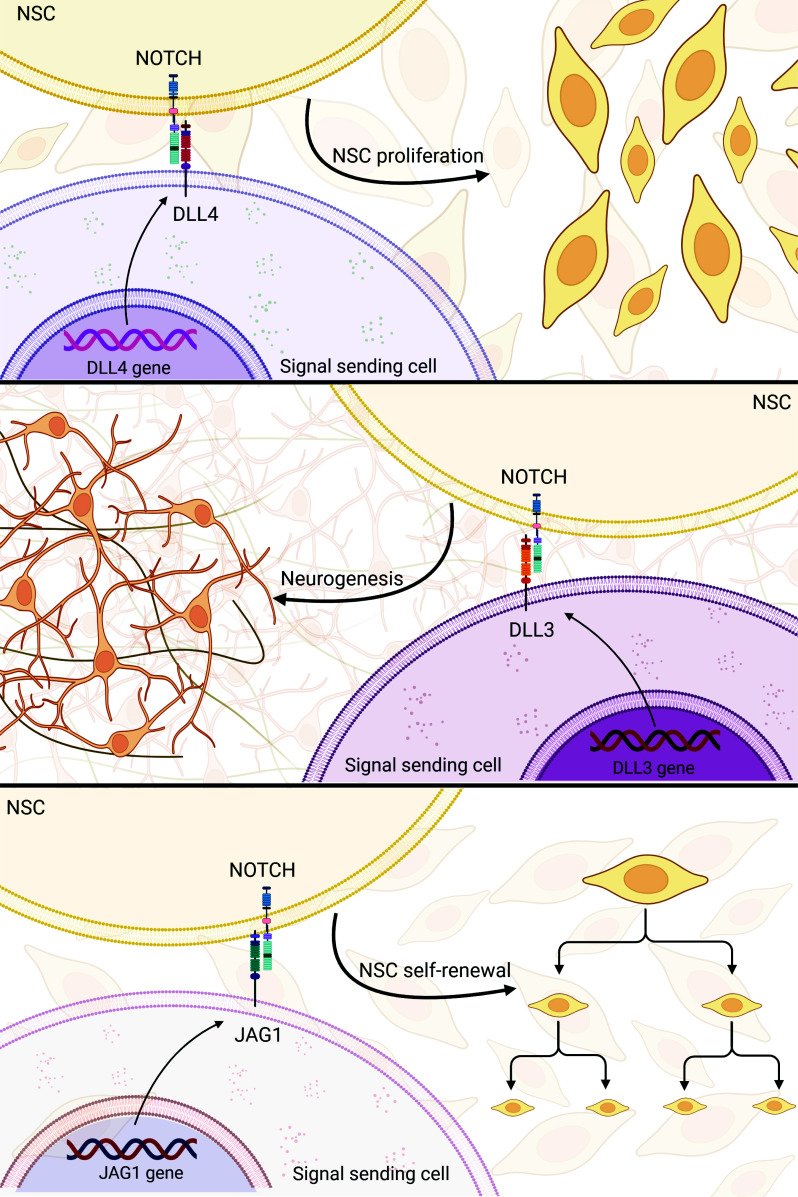
Modulation of Notch signaling in NSCs. Activation *via* DLL4 enhances NSC proliferation and progeny survival, leading to improved offspring outcomes. In contrast, DLL3 is not involved in NSC preservation but is crucial for guiding neuronal differentiation. Notch signaling through JAG1 promotes NSC self-renewal, contributing to maintaining the NSC population. (Created with BioRender.com).

MicroRNAs (miRNAs) are pivotal players in the developmental processes of neurons [87]. Qiao and colleagues demonstrated that NSC neurogenesis slowed as gliogenesis increased when NSCs were cultured *in vitro*. Moreover, miR-153 expression decreased. Further evidence that miR-153 promotes NSC neurogenesis comes from the observation that its overexpression induces neuronal differentiation while concurrently repressing glial differentiation in NSCs. Subsequent investigations revealed that miR-153 exerts regulatory control over neurogenesis by modulating the Notch pathway. Too much activity in the Notch signaling pathway causes a reduction in neurogenesis and an increase in gliogenesis [88]. miR-153 was shown to regulate the Notch signaling pathway simultaneously by targeting two critical components at distinct pathway stages. The diminished expression of Hes1 and Hey1 can be attributed to the inhibitory effect of miR-153, which reduces the levels of the JAG1 protein, thereby exerting control over the activity of the Notch signaling pathway [89] (Fig. **3**).

## 5 THE NOTCH SIGNALING PATHWAY IN ANIMAL MODELS OF MS

A widely utilized animal model for studying MS is experimental autoimmune encephalomyelitis (EAE) [90]. This model is established through the active immunization of animals using myelin antigens mixed with an adjuvant. Another approach to induce EAE involves the passive transfer of activated myelin-specific cellular clones or cell lines [91].

These intriguing findings emerged from studies utilizing the PLP/SJL EAE model. Pioneering research explored the effects of administering a compound known as GSI *via* oral and intraperitoneal routes. GSI, in this context, has the power to block Notch signaling in a broad, nonselective manner. The results were remarkable; a significant amelioration of clinical symptoms was observed, coupled with a notable reduction in the presence of Th1-associated cytokines. This finding illuminated the potential involvement of the Notch pathway in the progression of EAE [26]. Building upon this foundation, subsequent investigations delved deeper into the PLP/SJL EAE model. Their research revealed that another layer of complex GSI exhibited a remarkable ability to suppress the production of IL-17, a key player in the immune response. This finding strongly suggested the pivotal role of Notch signaling in the differentiation of Th17 cells, further enriching our understanding of the intricate mechanisms involved in MS research [92].

A study presented compelling evidence suggesting the significant involvement of Notch-3 in EAE. The researchers demonstrated a notable decrease in clinical illness scores and reductions in Th1 and Th17 cytokine levels. They achieved this by using a GSI that specifically targeted Notch-3 rather than Notch-1 [93].

Further exploration of the role of Notch-1 in macrophages revealed intriguing insights. Manipulating Notch-1 in these immune cells did not affect the Th1-type response in the EAE model. Instead, the cytokines released by transplanted macrophages seemed to disrupt the usual interaction between macrophages and T cells during disease induction [94].

Additionally, researchers have explored the impact of targeted deletion of Notch-1 in myeloid cells in mice. Their work revealed that blocking this receptor alleviates the symptoms of EAE. Consequently, there was less demyelination, reduced CD4+ T-cell infiltration, and decreased microglia/astrocyte activation. Moreover, isolation and characterization of CNS-infiltrated cells revealed a decrease in the presence of Th1 and Th17 cells [95].

The role of DLL4 in MS animal models has received greater attention than that of other Notch ligands.

Researchers have made intriguing observations regarding the expression of DLL4 on antigen-presenting cells (APCs). Their study revealed a significant increase in DLL4 expression in these cells. Interestingly, when DLL4 was blocked, clinical disease symptoms improved. Additionally, there was a notable decrease in the number of CD4+ T cells that produced both IFN-γ and IL-17 and a reduction in leukocyte infiltration into the CNS. Remarkably, this intervention had no discernible influence on the expression of Foxp3, a critical marker of the immune response. To explain these effects, researchers have proposed the following hypothesis: the inhibition of DLL4 leads to a reduction in CD4+ T-cell expression of the chemokine receptors CCR2 and CCR6. This, in turn, caused a shift in the migration and accumulation patterns of these cells within the CNS [96].

In a study utilizing the MOG/B6 EAE model, researchers achieved notable progress by inhibiting DLL4. This intervention led to a substantial reduction in the severity of clinical EAE. Furthermore, it orchestrated a transition in the immunological balance—from a predominant Th1/Th17 response to a Th2/Treg-mediated response—consistent with previous research findings. A significant finding arose when Tregs were depleted before EAE induction: Treg depletion nullified the protective effects of the anti-DLL4 mAb, indicating that DLL4 primarily regulates Treg formation [97].

In the initial phases of the EAE model, another study revealed an increase in DLL1 expression within dendritic cells (DCs). Intriguingly, blocking DLL1 led to a reduction in disease severity and a decrease in the number of CD4+IFN-γ+ cells. Conversely, engaging DLL1 through ligation produced the opposite outcome. Notably, these manipulations did not impact the frequency of CD4+Foxp3+ cells [98].

Further insights emerged from a study in the EAE C57BL/6J model, highlighting the role of B cells. B-cell activation led to an increase in Notch-1 expression. Remarkably, B cells themselves were found to express the Notch-1 ligands DLL1 and JAG1. This discovery has profound implications, suggesting that Notch-1, along with its ligands present in B cells, plays a pivotal role in promoting antibody production. This intricate interplay within the Notch-1 signaling pathway is crucial for antibody generation [99]. By expanding our scope to human research, another breakthrough was achieved. Researchers have employed a human JAG1 agonist peptide and revealed its role in ameliorating EAE progression. This improvement was closely correlated with an increase in the frequency of CD25+Foxp3+ T cells, offering hope for potential therapeutic interventions [100].

In the realm of MS research, the intricate role of JAG2-mediated signaling has been the focus of a study conducted by Elyaman and colleagues. Their research revealed the variable impact of JAG2 signaling on EAE, shedding light on the nuanced dynamics of this condition. This study demonstrated that Notch signaling is crucial for efficient IL-9 generation and is a key player in immune responses. When administered before antigen vaccination, JAG2 signaling molecules exert a beneficial effect. They enhance the development of IL-9-mediated regulatory T cells (Tregs) while reducing the severity of EAE. This finding indicates a potential avenue for therapeutic intervention. However, the scenario shifts when JAG2 signaling is introduced concurrently with immunization. In such cases, JAG2 signaling exacerbates the disease. This paradoxical effect may be attributed to the impact of IL-9 in the inflammatory milieu, which promotes the proliferation of Th17 cells—an aspect of the immune response associated with inflammation and autoimmunity [101].

The EAE model is not the only model used to examine Notch signaling; the results from these other models supplement what we know about this pathway in immune-mediated disorders.

Researchers aimed to investigate the potential impact of inhibiting Notch-1 on remyelination promotion by establishing a mouse model of demyelination. Their goal was to analyze myelin sheath restoration in the corpus callosum region through detailed microscopic analyses. The findings in the cuprizone-exposed group were notable. Positive myelin sheath staining significantly decreased, and the sheaths appeared sparsely stained with noticeably absent patches. However, different results were observed in different groups treated with CPZ or siNotch-1 (a molecule that inhibits Notch-1). Here, the myelin sheaths were denser and more organized. This outcome was noteworthy, as it indicated that the inhibition of Notch-1 significantly facilitated the recovery of mice during the remyelination phase. The impact was not limited to microscopic changes; it extended to the functional aspects of these mice. Those subjected to CPZ-induced acute demyelination experienced a substantial reduction in coordinated movement and balance abilities. However, this deficit was significantly attenuated in the groups receiving CPZ and siNotch-1 treatment. Returning to the microscopic realm, the researchers observed that myelinated axon density in the corpus callosum region was notably lower in both the CPZ group and the CPZ + siRNA NC (a control group) group than in the control group. However, the control group and the control + siNotch-1 and control + siRNA NC groups exhibited intact myelin sheath structures with uniform thickness. The axons were relatively complete, and many myelin-embedded axons were evident. Importantly, there were no pathological changes, such as mitochondrial edema [102].

In a comprehensive study aimed at understanding the dynamics of the inflammatory response during demyelination and subsequent remyelination, researchers investigated the impact of CPZ treatment on male and female rats. Their investigation provided crucial insights into the changes occurring within the nervous system. One of the key findings of this study was a noticeable reduction in myelin proteins, including myelin basic protein (MBP) and myelin-associated glycoprotein (MAG), among rats treated with CPZ compared to their control counterparts. The most extensive demyelination was observed on day 0, marking the initial phase of the study. However, there was gradual myelin content recovery over time, with levels nearly reaching those of the control group by day 21. Changes in cell populations characterize the inflammatory response in the context of demyelination and remyelination. Researchers have noted an elevated proportion of cells that stain positive for GFAP, ionized calcium-binding adapter molecule 1 (Iba-1), and CD68. These findings indicated an expansion in the microglial population in response to injury. Notably, phagocytic microglia were exclusively observed in CPZ-treated animals, highlighting their role in clearing myelin debris.

Furthermore, this study explored JAG1-mediated Notch signaling during the injury-induced demyelination-remyelination process. Researchers observed a notably larger portion of cells expressing JAG1 on both day 7 and day 0 in CPZ-treated rats. Interestingly, these JAG1-expressing cells were GFAP-positive, while none were NG2- or Olig2-positive. Additionally, this study investigated the role of OPCs in myelin repair postinjury. The proportion of NG2 cells was significantly greater in the CPZ-treated group than in the control group on day 0. However, there was a significant decrease in the proportion of NG2 cells between days 7 and 14 under both conditions, indicating a dynamic response of these cells to demyelination and remyelination [103].

Table **1** summarizes various findings related to Notch signaling in animal models of MS and demyelination.

## 6 NNOTCH SIGNALING AS A POTENTIAL THERAPEUTIC TARGET FOR MS

As described above, chronic MS lesions contain OPCs, which can develop and mitigate the harmful consequences of remyelination [104]. Nevertheless, it is important to note that the Notch signaling pathway exerts an inhibitory influence on the development of these cells. For the first time, in 2002, it was demonstrated that both in cell culture and in the brains of MS patients, due to the increase in TGF-β, the upregulation of JAG1 expression on active astrocytes suppresses oligodendroglial differentiation *via* Notch-1- and Hes5-dependent pathways [105]. This theory was confirmed by further research using Notch signaling inhibitors in MS animal models. Therefore, pharmacological inhibition of the Notch pathway seems promising for promoting remyelination in MS lesions due to the presence of OPCs in MS patients.

However, findings from other researchers propose an alternative perspective indicating that Notch signaling may not hinder remyelination; instead, it might foster this process. Previous studies have demonstrated that the Notch ligand contactin on the surface of demyelinated axons facilitates remyelination through γ secretase-dependent nuclear translocation of the NICD [59]. This pathway safeguards the OPC pool and modifies OL development by conveying localized ligand- or receptor-specific environmental cues from demyelinated axons [106].

To date, no human clinical trials have indicated whether the participation of Notch in remyelination constitutes a potential MS treatment. However, some FDA- and EMA-approved drugs for MS and other disorders have been shown to benefit resident CNS cells, such as OPCs, and their immunomodulatory effects. For example, benztropine, an anticholinergic molecule, inhibits parasympathetic nerve activation and is approved for Parkinson’s disease treatment [107]. This drug activates muscarinic receptors, which in turn enhances remyelination (in a noninflammatory demyelination model induced by cuprizone) and reduces clinical severity in EAE models; it also stimulates OPC differentiation by inhibiting Notch signaling [108]. Further research is required to determine the efficacy of benztropine and its underlying mechanisms.

Quercetin is a flavonoid molecule that inhibits intramembranous γ-secretase and has not yet been approved. It inhibits the canonical Notch signaling pathway and improves remyelination and clinical recovery in the EAE model [107].

Broad-spectrum inhibitors or activators of the Notch signaling cascade may lack precision and effectiveness. Instead of exclusively addressing the central Notch pathway, a more favorable approach would involve targeting factors that directly interact with or regulate this pathway within specific disease scenarios. This endeavor would require a combination of extensive genetic screening, computational network analyses, and intricate mechanistic investigations to pinpoint these influential factors. The developmental consequence of Notch signal suppression or activation is highly cell context-dependent and thus impossible to anticipate in advance [109]. The complexity of this pathway requires extreme caution, and this research will be a great challenge but one that will be worthy of further study.

## CONCLUSION

The Notch signaling pathway is assumed to be a functional regulator of the growth and specialization of numerous cellular varieties within the CNS. Remarkably, a substantial impact is exerted on determining the destiny of neural progenitor cells, especially during the switch from neuronal to glial pathways.

Significantly, microglia, the immune cells residing in the CNS, have recently attracted attention in studies of the Notch signaling pathway. Investigations indicate that this pathway has an immersive impact on microglial growth and stimulation, influencing neuroinflammatory reactions. Nevertheless, differing discoveries have revealed evidence of the interplay of Notch with other signaling pathways due to the intricate nature of these associations.

In astrocytes, Notch signaling activation in neuronal precursors and fledgling neurons encourages methyl group removal and the activation of astrocyte-specific genes. This process occurs through the JAK-STAT pathway and is facilitated by cytokines. The interaction of Notch with cytokine signaling plays a crucial role in influencing the equilibrium between neurons and glial cells in CNS development, with possible consequences for neural function.

Within the oligodendrocyte lineage, OL differentiation and myelination timing are regulated by Notch signaling. This process is due to dynamic influences from the interactions of OLs with their microenvironment, which, in turn, impacts the expression of proteins related to Notch signaling. These interactions involve the expression of Notch ligands such as JAG1 and facilitate OL differentiation. This finding underscores the context-dependent nature of Notch signaling in myelin development.

NG2 cells, which function as progenitors for oligodendrocytes, differentiate into astrocytes, neurons, or mature oligodendrocytes under the regulation of Notch signaling. The regulation of NG2 cell fate involves Notch ligands and downstream mechanisms, thereby developing the CNS and pathological conditions such as CNS injury and remyelination.

The intricate involvement of Notch signaling in CNS development, neurogenesis, and its potential therapeutic implications for multiple sclerosis (MS) is well known. Experiments have shown that Notch signaling plays a pivotal role in regulating neural stem cells, their differentiation processes, and overall CNS development. Furthermore, its impact on animal models of MS provides perspectives for potential therapeutic interventions.

Ongoing research will demonstrate whether to promote or inhibit remyelination by regulating Notch signaling pathways in MS. Some studies suggest that remyelination could be augmented through Notch inhibition, influencing precursor cells of oligodendrocytes. Conversely, alternative viewpoints show that Notch signaling may facilitate this process.

In exploring these complex interactions, the significance of precision emerges as a crucial factor considering the employment of Notch signaling as a target for MS treatments. Diverse cell types, signaling molecules, and the specific circumstances of these interactions demand a well-organized strategy. Findings from studies on medications such as benztropine and quercetin, which modulate Notch signaling and control the process of remyelination, have inspired optimism for positive therapeutic approaches.

The Notch signaling pathway in the CNS forms a network, highlighting its important involvement in neural development, differentiation, and potential aspects for therapy, especially in MS treatment. Therefore, understanding the interactions among different cell types and signaling molecules is crucial for investigating therapeutic options and modulating the Notch signaling pathway.

## Figures and Tables

**Fig. (1):**
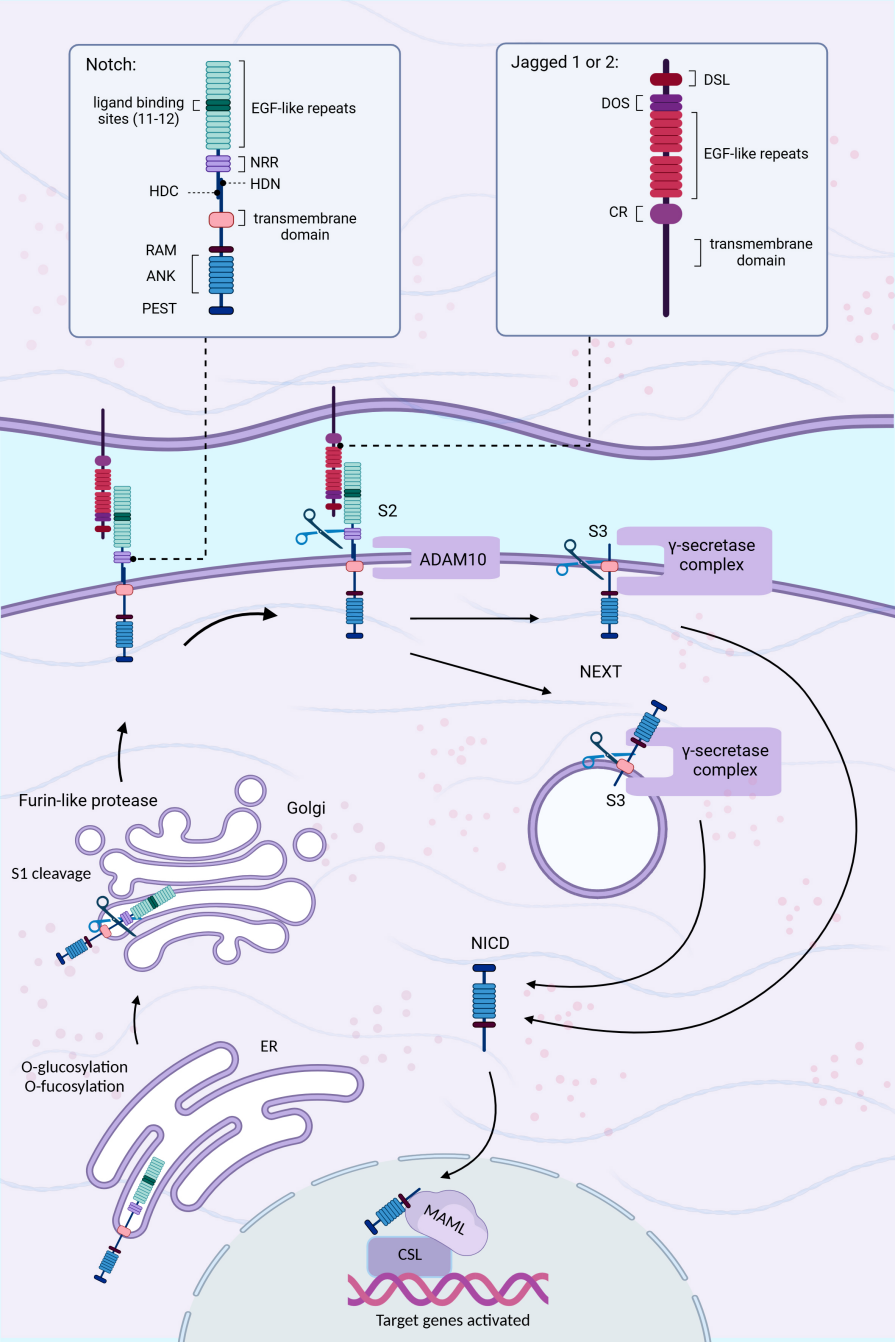
The Notch signaling pathway. In this schematic representation, the Notch receptor precursor is initially synthesized within the ER and undergoes glycosylation in the EGF-like repeat domain. Subsequently, it is transported to the Golgi apparatus, where the first proteolytic cleavage, S1, occurs, forming the mature Notch heterodimer. Five recognized Notch ligands in humans (DLL1, DLL3, DLL4, JAG1, and JAG2) trigger a conformational change in Notch upon attachment. This exposes the cleavage site for the ADAM10 protease, releasing the Notch extracellular truncation (NEXT) segment. Further cleavage at the S3 site by the γ-secretase complex liberates the Notch intracellular domain (NICD), which translocates to the nucleus. NICD interacts with CSL and MAML within the nucleus to activate downstream gene expression, representing a pivotal step in Notch signaling. Two models, endocytic-activation and endocytosis-independent, describe the location of S3 cleavage. (Created with BioRender.com).

**Fig. (2):**
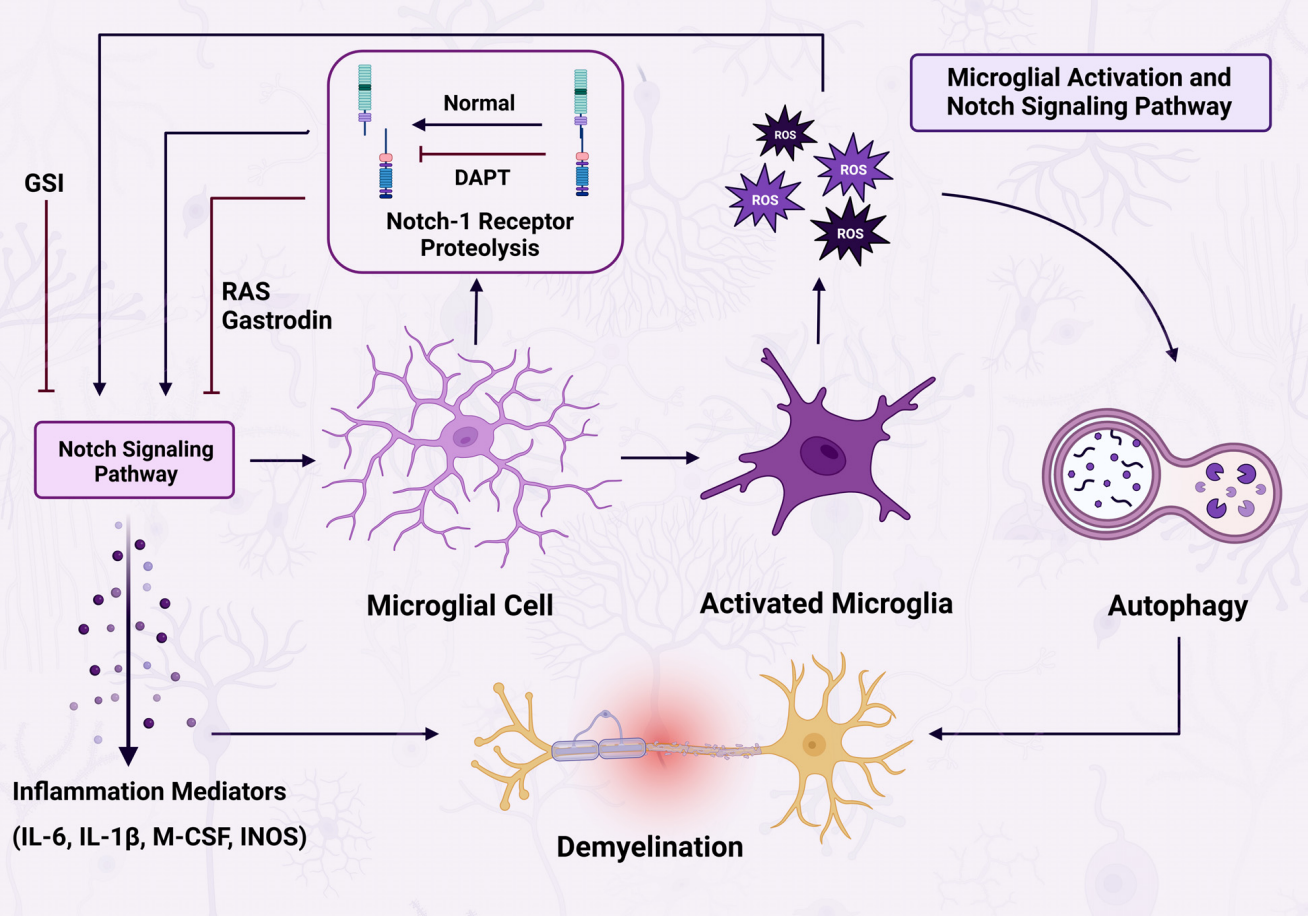
The role of Notch signaling and microglial activation in demyelination. The Notch signaling pathway has emerged as a central protagonist of neuroinflammation. γ Secretase inhibitors (GSIs) have been shown to mitigate demyelination in multiple sclerosis (MS). Microglia express Notch-1 in demyelinated regions. Coordinated modulation of Notch-1 signaling, which is driven by the RAS pathway and gastrodin, causes inflammation. In postnatal rats, elevated Notch-1, TNF-α, and IL-1β in activated microglia have been demonstrated. DAPT, a Notch cleavage inhibitor, reduces cytokine levels but paradoxically increases Notch-1 labeling. DAPT also suppresses microglial activation and cytokine production. Notably, inhibiting Notch-1 increases proinflammatory cytokines and enhances phagocytosis. These paradoxes suggest complex interactions within cellular signaling pathways. This finding demonstrates the intricate role of Notch signaling in neuroinflammation. (Created with BioRender.com).

**Table 1:** Notch signaling-related findings and their impact on disease outcomes in different animal models of MS.

**Study**	**Animal Models**	**Notch-related Findings**	**Impact on Disease**	**References**
Minter *et al*.	PLP/SJL EAE	GSI blocked Notch signaling nonspecifically	Improved clinical symptoms, decreased Th1 cytokines	[26]
Keerthivasan *et al*.	PLP/SJL EAE	Implication of Notch in Th17 Differentiation	GSI suppresses IL-17 production	[92]
Jurynczyk *et al*.	PLP/SJL EAE	Notch-3 targeted GSI reduced clinical illness	Reduced Th1 and Th17 cytokine levels and a notable decrease in clinical illness scores	[93]
Wongchana *et al*.	MOG/B6 EAE	Manipulating Notch-1 in macrophages did not impact the Th1 response	Disrupted macrophage-T-cell interaction	[94]
Fernلndez *et al*.	MOG/B6 EAE	Blocking Notch-1 alleviated EAE symptoms	Reduced demyelination, less microglia/astrocyte activation, T-cell infiltration, Th1/Th17 cells	[95]
Reynolds *et al*.	PLP/SJL EAE	DLL4 inhibition improved clinical disease	Reduced CD4+ T cells producing IFN-𝛾 and IL-17, reduced leukocyte infiltration.	[96]
Bassil *et al*.	MOG/B6 EAE	DLL4 inhibition shifted response to Th2/Treg	DLL4's role in Treg formation, decrease in the severity of clinical EAE	[97]
Elyaman *et al*.	MOG/B6 EAE	DLL1 manipulation impacted disease severity	Reduced CD4+IFN-𝛾+ cells	[98]
Zhu *et al*.	MOG/B6 EAE	Notch-1 on B cells promoted antibody production	Notch-1 signaling is crucial for antibodies	[99]
Palacios *et al*.	MOG/B6 EAE	JAG1 signaling correlated with increased CD25+Foxp3+ T cells	Improved EAE progression	[100]
Elyaman *et al*.	MOG/B6 EAE	Decreased EAE with JAG2 Signaling Prior to Antigen Vaccination	increase IL-9-mediated Treg-cell development	[101]
Elyaman *et al*.	MOG/B6 EAE	Aggravation of Disease with JAG2 Signaling During Simultaneous Immunization	aggravate the disease because IL-9 favors the proliferation of Th17 cells in an inflammatory environment	[101]
Fan *et al*.	Cuprizone model	Inhibition of Notch-1 facilitated remyelination	Improved recovery in mice during remyelination	[102]
Mathieu *et al*.	Cuprizone model	JAG1-expressing cells increased during injury	Implication of JAG1 in response to injury	[103]
Mathieu *et al*.	Cuprizone model	NG2 cells increased initially, then declined	Involvement of NG2 cells in myelin repair	[103]

## LIST OF ABBREVIATIONS

AMCs: Amoeboid Microglia
ANK: Ankyrin Repeat
bHLH: Basic Helix-loop-helix
CNS: The Central Nervous System
CNTF: Ciliary Neurotrophic Factor
CT-1: Cardiotrophin-1
DLL1: Delta-like Ligand 1
DLL3: Delta-like Ligand 3
DLL4: Delta-like Ligand 4
ECD: Extracellular Domain
ER: Endoplasmic Reticulum
GSI: γ-secretase Inhibitors
JAG1: Jagged-1
JAG2: Jagged-2
LIF: Leukemia Inhibitory Factor
LNRs: Lin12-Notch repeats
LPS: Lipopolysaccharides
MAML: Mastermind-like
MS: Multiple Sclerosis
NEXT: Notch Extracellular Truncation
NGN-1: Neurogenin-1
NICD: Intracellular Domain
NO: Nitric Oxide
NPCs: Neural Progenitor Cells
NRR: Negative Regulatory Region
NSCs: Neural Stem Cells
OLPs: Oligodendrocyte Progenitor’s Cells
OLs: Oligodendrocytes
OPCs: Oligodendrocyte Progenitor Cells
RAM: RBPJ Association Module
SCC: Spinal Cord Contusion
SV: Subventricular Zone
VZ: Ventricular Zone
